# Mild Cognitive Impairment: Statistical Models of Transition Using Longitudinal Clinical Data

**DOI:** 10.1155/2012/291920

**Published:** 2012-03-25

**Authors:** Erin L. Abner, Richard J. Kryscio, Gregory E. Cooper, David W. Fardo, Gregory A. Jicha, Marta S. Mendiondo, Peter T. Nelson, Charles D. Smith, Linda J. Van Eldik, Lijie Wan, Frederick A. Schmitt

**Affiliations:** ^1^Sanders-Brown Center on Aging, University of Kentucky, Lexington, KY 40536, USA; ^2^Alzheimer's Disease Center, University of Kentucky, Lexington, KY 40536, USA; ^3^Department of Biostatistics, University of Kentucky, Lexington, KY 40536, USA; ^4^Department of Statistics, University of Kentucky, Lexington, KY 40536, USA; ^5^Department of Pathology, University of Kentucky, Lexington, KY 40536, USA; ^6^Department of Anatomy and Neurobiology, College of Medicine, University of Kentucky, Lexington, KY 40536, USA; ^7^Department of Neurology, College of Medicine, University of Kentucky, Lexington, KY 40536, USA

## Abstract

Mild cognitive impairment (MCI) refers to the clinical state between normal cognition and probable Alzheimer's disease (AD), but persons diagnosed with MCI may progress to non-AD forms of dementia, remain MCI until death, or recover to normal cognition. Risk factors for these various clinical changes, which we term “transitions,” may provide targets for therapeutic interventions. Therefore, it is useful to develop new approaches to assess risk factors for these transitions. Markov models have been used to investigate the transient nature of MCI represented by amnestic single-domain and mixed MCI states, where mixed MCI comprised all other MCI subtypes based on cognitive assessments. The purpose of this study is to expand this risk model by including a clinically determined MCI state as an outcome. Analyses show that several common risk factors play different roles in affecting transitions to MCI and dementia. Notably, APOE-4 increases the risk of transition to clinical MCI but does not affect the risk for a final transition to dementia, and baseline hypertension decreases the risk of transition to dementia from clinical MCI.

## 1. Introduction

Mild cognitive impairment (MCI) often refers to the clinical condition between normal cognition and probable Alzheimer's disease (AD). However, persons diagnosed with MCI may progress to non-AD forms of dementia, remain MCI until death, and in some instances recover to a normal cognitive state [1–3]. There has been considerable effort to refine diagnostic criteria, separate MCI into amnestic and nonamnestic subtypes, and identify the underlying etiologies of MCI [1, 4, 5]. However, whether MCI is a true precursor to dementia remains controversial [6–9] despite evidence of AD neuropathology in amnestic MCI [10, 11]. This is due in part to the description of “back transitions” (i.e., recovery to normal cognition) that have been reported in longitudinal studies [3, 9, 12, 13]. Although the long-term prognosis for such cases is unclear, patients with a Clinical Dementia Rating (CDR) global score of 0.5 often have AD pathology at autopsy regardless of back transitions to CDR global scores of 0 [14]. Back transitions are likely heterogeneous in origin and may be explained by misclassification of either the MCI or normal state, interclinician differences in application of diagnostic criteria, within-patient variability due to medical illness or psychosocial factors, or resistance to cognitive decline due to cognitive reserve [15–18].

In a previous study we investigated MCI as defined by cognitive test performance alone. Here, we have added a clinical consensus-based MCI state as defined by the Second International Working Group on MCI [1] and operationalized by the National Alzheimer's Coordinating Center (NACC) and the Alzheimer's Disease Neuroimaging Initiative (ADNI) [19, 20]. We now have sufficient data on this MCI state to assess it as risk factor for dementia. The purpose of this study is to describe our statistical model of longitudinal data in the context of studying MCI risks and to update our prior research with additional cognitive assessments and clinical diagnoses from a large longitudinal sample. Over 54% of the sample subjects now have a terminating event (i.e., we have 35 additional dementias and 69 additional deaths) compared to the 36% in the previous study. These additional events provide increased power to detect potential risks for transition including age, gender, education, APOE-4, family history of dementing illness, and baseline hypertension.

## 2. Methods

### 2.1. Subjects

Subjects in the current study are from the Biologically Resilient Adults in Neurological Studies (BRAiNS) at the University of Kentucky's Alzheimer's Disease Center (UK ADC), a longitudinal cohort of 1,030 individuals with ongoing recruitment established in 1989 [21]. Participants consent to extensive annual cognitive and clinical examinations as well as brain donation upon death. Exclusion criteria include age less than 60 years, active infectious diseases, neurological disorders, psychiatric disorders, disabling medical disorders, and dementing illness. Subjects included in the current study (*n* = 554) comprise those included in the previous report [22]. All subjects were cognitively intact at study entry. All research activities were approved by the University of Kentucky Institutional Review Board. Each participant gave written informed consent.

### 2.2. Cognitive Assessments

Annual cognitive test-based assessments taken on a cohort of initially cognitively normal subjects participating in the BRAiNS project are used to classify subjects into one of three states: normal, test-based amnestic MCI (aMCI_TB_), or test-based mixed MCI (mMCI_TB_) (Table 1). Classification of aMCI_TB_ and mMCI_TB_ has been described previously [22, 23]. Briefly, a classification of aMCI_TB_ results from a poor score (as defined below) on at least one measure of episodic memory measure (Table 1). A classification of mMCI_TB_ requires a poor score on at least one measure of language or executive function (Table 1) regardless of the aMCI_TB_ classification status. A poor score is defined as at least 1.5 standard deviations (SD) below the age-adjusted mean, which is consistent with the Second International Working Group on MCI criteria [1]; normative values were derived from the baseline evaluations of the entire normal cohort.

Classification into clinical consensus-based MCI (MCI_CC_) results from a diagnosis of MCI, which is determined according to the consensus guidelines on MCI developed by the Second International Working Group on MCI [1]. A diagnosis of MCI requires

1. a cognitive complaint by the subject or informant, or evidence for longitudinal decline on cognitive test performance (at least 1.5 SD decline);
2. generally intact global cognition;
3. no or minimal functional impairment;
4. not demented by DSM-IV criteria.

Additionally, MCI_CC_ is restricted to those individuals for whom a neurodegenerative etiology is suspected. The NACC diagnostic criteria designate patients with cognitive impairments but without a presumed degenerative etiology as “cognitive impairment, not MCI” [19]. Diagnosis of MCI_CC_ is based on a consensus team review by the examining physician, neuropsychologist, and the clinical research assistant administering the protocol [13]. This MCI_CC_ designation is equivalent in most respects to the new “MCI-Core Clinical Criteria” as defined by the National Institute on Aging-Alzheimer's Association Workgroup on Diagnostic Guidelines for Alzheimer's Disease [24]. The primary difference is that the new criteria allow the cognitive complaint in number one above to come from a skilled clinician rather than only the patient or informant. A dementia classification also results from a clinical consensus diagnosis of dementia (most often AD), which may be based on the dementia criteria of Diagnostic and Statistical Manual of Mental Disorders Fourth Edition (DSM-IV) [25], criteria of the Joint Working Group of the National Institute of the Neurologic and Communication Disorders and Stroke-AD and Related Disorders (NINCDS-ADRDA) [26], NINDS-AIREN criteria for vascular dementia [27], and the 2005 Dementia with Lewy bodies (DLB) Consortium revised criteria [28]. A diagnosis of MCI_CC_ or dementia supersedes a classification of normal cognition, aMCI_TB_ or mMCI_TB_ in our model.

Between their annual assessments, subjects may die or become demented, and these states are treated as completely absorbing competing states. MCI_CC_ is treated as a quasi-absorbing state, as subjects do not move backward to a transient state (i.e., normal cognition, aMCI_TB_, or mMCI_TB_), but they may become demented or die.

For 19 subjects, review of the longitudinal record revealed apparent back transitions from MCI_CC_ to normal: nine subjects were diagnosed with MCI_CC_, reverted to normal, and then reconverted to MCI_CC_, three of whom eventually became demented; six subjects had a single diagnosis of MCI_CC_ between several diagnoses of normal cognition on either side; and four subjects had a single diagnosis of MCI_CC_ at their initial evaluation following the UK ADC's implementation of the NACC Uniform Data Set (UDS) cognitive and clinical testing protocol [19, 29] with all subsequent evaluations classified as normal. Review of each subject's complete study history revealed in all cases that the apparent back transitions were the result of underlying medical conditions, conflicting data from informants, or misclassification. Given that there are differences in the medical comorbidities (e.g., hypothyroidism, B_12_ deficiency) that can mimic MCI_CC_ in both research and general clinic settings (cf., [13]), “treatable” cases of MCI_CC_ were not considered to reflect neurodegenerative conditions. Similarly, a single diagnosis of “normal” in the midst of many years of MCI_CC_ diagnoses appears to reflect a temporary resolution of a neurodegenerative condition and so strains credulity. Therefore, in light of the available evidence, the six normal to MCI_CC_ to normal cases and the four MCI_CC_ to normal cases were reclassified as never having MCI_CC_, though they still might be classified aMCI_TB_ or mMCI_TB_, and the nine MCI_CC_ to normal to MCI_CC_ were reclassified as MCI_CC_ at every assessment after the first diagnosis of MCI_CC_.

### 2.3. 2.3. Statistical Analysis

**Figure 1:**
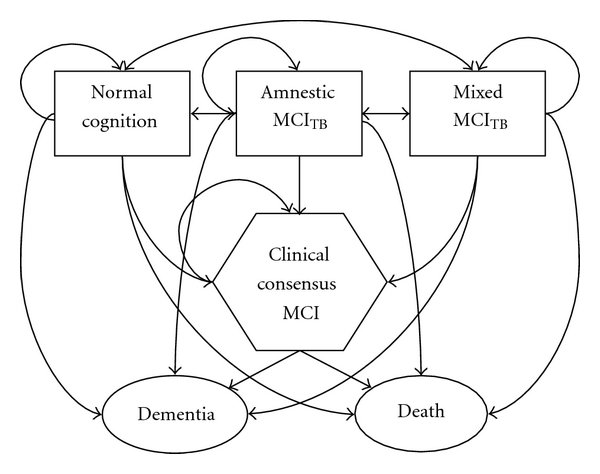
Flow diagram of transitions possible between subject visits. Normal cognition is the base state for transitions made from normal cognition, test-based amnestic MCI, and test-based mixed MCI; clinical consensus MCI is the base state otherwise.

The conditional distribution of the cognitive status at any assessment given the status at the prior assessment is assumed to have the Markov property. That is, the status at the current assessment depends only on the status at the prior assessment [30] and possibly other risk factors. A multistate Markov chain with three transient states (normal cognition, aMCI_TB_, and mMCI_TB_), one quasi-absorbing state (MCI_CC_), and two absorbing states (death and dementia) was used to model the probability of maintaining the current state or moving to a different state at the next assessment (Figure 1). The Markov chain models the log-odds of transition between any two temporally adjacent assessments, here called the “prior state” and the “current state”, versus remaining in or returning to a “base state” with a series of four random effects polytomous logistic regression models (i.e., one model for each transient state and one model for the quasi-absorbing state, MCI_CC_).

The base state is normal cognition while a participant's prior state is normal cognition, aMCI_TB_, or mMCI_TB_; once a participant has moved into MCI_CC_, the base state then becomes MCI_CC_. The model is additive, which means in practice that although we assume the risk factors are independent of the *prior state* (i.e., the effect of sex, e.g., is the same whether the prior state is normal cognition, aMCI_TB_, or mMCI_TB_; there is no interaction between the covariates and the prior state), the estimated risk factor beta coefficients may depend on the *base state*. That is, the effect of sex, for example, may vary with respect to a base state of normal cognition versus a base state of MCI_CC_. To account for within-subject correlations, a normally distributed shared random effect due to Salazar et al. [31] was included in the model using PROC NLMIXED in SAS 9.2 (SAS Institute Inc, Cary, NC). The Quasi-Newton method is used to maximize the likelihood function, which due to the presence of the shared random effect is an integral approximated by an adaptive Gaussian quadrature with one quadrature point [32, 33]. Transitions to MCI_CC_ and dementia states are assumed to have occurred on the date of assessment as modeling assumptions do not permit the inclusion of interval censoring-type approaches. The model ignores any transitions among the transient states between regularly scheduled assessments. Statistical significance was set at *α* = 0.05.

### 2.4. Covariates

Covariates of interest include age at assessment (centered at 78, the sample median), sex (1 = female, 0 = male), education (two levels: ≤12 years, >12 years), presence (1) or absence (0) of any copies of the APOE-4 allele, presence (1) or absence (0) of family history of dementing illness among first degree relatives, and presence (1) or absence (0) of hypertension at study entry. Hypertension status at entry was derived from participant responses to the question “have you ever been told by a doctor or nurse that you have high blood pressure?” Use of medications was also recorded; however, reported use of an antihypertensive medication did not supersede a participant's response of “no” since anti-hypertensives are used to treat other illnesses. Also included as covariates (when the base state is normal cognition) are two indicator variables for (1 = yes, 0 = no) aMCI_TB_ and mMCI_TB_; normal cognition is the reference category. Race was not included as a covariate because almost all of the included subjects (99%) are Caucasian.

## 3. Results

Study participants contribute an average of 10.8 annual assessments (median = 10 assessments, mode = 10 assessments) with the average time between assessments at approximately 13 months (Table 2). Approximately 87% of subjects who reported hypertension at baseline also reported taking at least one anti-hypertensive medication, whereas 15% of those who reported no history of hypertension reported taking at least one anti-hypertensive medication.

### 3.1. One-Step Transitions

Table 3 enumerates the one-step transitions associated with each arrow in Figure 1. The majority of transitions from aMCI_TB_, which requires a poor score on a test of episodic memory, are back to normal cognition at the next visit (59.3%), and only 4.4% are transitions to MCI_CC_ or dementia. Mixed MCI (mMCI_TB_), which requires a poor score on a test of executive function or language, appears more predictive of underlying impairment with 43.8% remaining mMCI_TB_ and 7.1% transitioning to MCI_CC_ or dementia at the next visit. Entry into MCI_CC_ is a clear risk factor for transition to dementia since the majority of the transitions into the dementia state come from MCI_CC_ when compared to transitions into dementia from the other states. As previously stated, recovery from MCI_CC_ does not occur. We note that 13 of the 16 subjects who were MCI_CC_ and died without a dementia diagnosis have been autopsied. Of these, five had AD-type pathology insufficient for an AD diagnosis, two had mixed AD and vascular pathology, two had mixed vascular pathology (one with Lewy bodies and one with hippocampal sclerosis), two had hippocampal sclerosis, one had Parkinson's disease, and one had no histopathologic substrate for dementia (see also Reference [34]).

### 3.2. Risk Factors

A number of risk factors alter the probability of transition to an MCI state (Table 4). Older age increases the risk of movement into aMCI_TB_ (*P* = 0.0006) and mMCI_TB_ (*P* < 0.0001). In addition, 12 (or fewer years) of education predicts transition to mMCI_TB_ (*P* = 0.0001) but not aMCI_TB_. Family history of dementia “protects” against transitions to mMCI_TB_ (*P* = 0.011), and female sex is protective against entry into aMCI_TB_ (*P* = 0.013). Classification as mMCI_TB_ at the prior assessment is predictive of remaining mMCI_TB_ rather than returning to normal at the next assessment (*P* < 0.0001).

Demographic risk factors for transition to the MCI_CC_ state (versus remaining in or returning to a normal state) are older age (*P* < 0.0001), presence of at least one APOE-4 allele (*P* = 0.0053), and high school education (12 years) or less (*P* = 0.007). Classification as either aMCI_TB_ or mMCI_TB_ at the prior assessment also increases the risk of transition to MCI_CC_ (*P* = 0.0041 for aMCI_TB_, *P* < 0.0001 for mMCI_TB_).

In the absence of MCI_CC_, risk factors for dementia include older age (*P* < 0.0001) and the presence of at least one APOE-4 allele (*P* = 0.0057) (Table 4). A classification as mMCI_TB_ (*P* < 0.0001) but not aMCI_TB_ at the prior assessment also increases the risk of transition to dementia at the next visit. Risk factors for transition to death without dementia include older age (*P* < 0.0001) and self-reported hypertension at study entry (*P* = 0.018).

Participants in this sample who transitioned from MCI_CC_ to dementia (*n* = 34) did so in an average of 2.5 ± 1.5 years (median = 2.2 years), and those who transitioned from MCI_CC_ to death without an intervening dementia (*n* = 16) did so in an average of 2.7 ± 1.7 years (median = 3.4 years). Those cases that remain in the MCI_CC_ state (*n* = 50) have carried the diagnosis for an average of 4.1 ± 2.4 years (median = 4.2 years). Once a transition to MCI_CC_ has occurred, only history of hypertension at study entry appears to influence further transitions to dementia, or death without dementia, versus remaining in the MCI_CC_ state (Table 5). A participant who reported baseline hypertension is more likely to remain MCI_CC_ (*P* = 0.037) than to convert to dementia at the next visit: the yearly transition rate to dementia for those with hypertension at baseline is approximately 4.2% and 12.6% for those without hypertension at baseline.

## 4. Discussion

The addition of the MCI_CC_ state to the multistate Markov chain confirms the utility of cognitive testing in predicting true underlying cognitive impairment. Entry into aMCI_TB_ and particularly mMCI_TB_, both of which are determined solely by poor performance on cognitive assessment, increases the risk of a diagnosis of MCI_CC_ at the next visit versus returning to normal. These results highlight the importance of objective criteria in MCI diagnosis and emphasize the role of cognitive testing, particularly of language and executive function, in early detection. Notably, poor performance limited to tests of episodic memory (aMCI_TB_) in this population can resolve to normal performance at the next annual assessment as much as 60% of the time and progress to MCI_CC_ just 3% of the time (Table 3). Poor performance on tests of language and executive function is somewhat more stable, returning to normal performance at the next annual assessment 33% of the time. While there is no question that MCI_TB_ predicts MCI_CC_, these findings emphasize that clinicians who primarily rely on cognitive testing should obtain longitudinal followup before a diagnosis of MCI is given to the patient [35].

These findings reflect a novel analysis of risk factors for MCI and dementia based on the current NACC UDS criteria that are used across AD centers in the United States [19] and so represent a standardized diagnostic system in contrast to earlier analyses of MCI risk factors [36, 37]. Further, the comparison of two different sets of MCI criteria (MCI_CC_ versus MCI_TB_) provides differing risk factors that could be of clinical use in patient care. This is best highlighted in our group's earlier comparison of patients diagnosed with MCI in a clinical research (i.e., the UK ADC BRAiNS cohort) as well as a memory clinic setting where only 9% of patients in the memory clinic had nonneurodegenerative causes for cognitive decline in contrast to 31% of the research clinic cases [13].

Risk factors for one-step transitions into MCI_CC_ include age, low education, prior classification as either aMCI_TB_ or mMCI_TB_, and the presence of at least one APOE-4 allele. APOE-4 is a known risk factor for AD, and although results for MCI have been mixed, a recent study of a nationally representative sample reported that APOE-4 was a reliable predictor of MCI versus normal cognition [38], and data from the Religious Orders Study reveal a 1.4-fold increased risk of MCI in persons with an APOE-4 allele [39].

It is clear that once an individual has transitioned to MCI_CC_, the risk of dementia increases dramatically. In this sample, 38.5% of individuals with MCI_CC_ have transitioned to dementia (at an estimated overall rate of 12.6% per year) compared to 11.8% of individuals with no history of MCI_CC_ (at an estimated overall rate of 0.16% per year). However, common risk factors for dementia (i.e., age, sex, education, family history, and APOE-4) do not predict whether an individual will remain in MCI_CC_ or transition to dementia, or death without dementia, at the next visit. Similar results have been reported in studies that have examined risk factors for progression of cognitive impairment. Tschanz et al. [40] noted in the Cache County cohort that while female sex and age at onset were predictive of decline in Mini-Mental Status Exam (MMSE) scores, education was not related to rate of MMSE decline, and APOE-4 was related to earlier onset of impairment but not rate of MMSE decline. Fleisher et al. [41] reported that although APOE-4 did predict conversion from amnestic MCI to AD over a 36-month interval, it did not improve the predictive accuracy of their model (which included only neuropsychological test scores).

Participants who reported hypertension at baseline were significantly less likely to transition from MCI_CC_ to dementia at the next visit, which may indicate a primarily vascular rather than an AD or mixed AD and vascular etiology for MCI_CC_ in these patients. Several studies have shown that brain white matter changes are associated with cognitive decline in aging [42, 43] and that vascular changes exacerbate the cognitive decline associated with AD [44, 45]. Hoffman and colleagues [46] reported that autopsied subjects who took anti-hypertensive medications had significantly less Alzheimer-type pathology than either those with no history of hypertension or those with hypertension not treated by medication. However, the differences in risks for treated and untreated hypertension could not be assessed here due to the small number of cases of untreated hypertension in the sample.

As with aMCI_TB_, mMCI_TB_, and MCI_CC_, older age increases the probability of a transition to a dementia state. Baseline hypertension plays no role in transitions to aMCI_TB_, mMCI_TB_, MCI_CC_, or dementia (in the absence of MCI_CC_), predicting only transitions to death (modeled as a competing risk for dementia). This result agrees with our previous research [23] even after four additional years of followup, as well as with the results of a recent meta-analysis, which found no increased risk of incidence of AD for either persons with hypertension or those taking anti-hypertensive medications [47]. We note that hypertension is a time-dependent risk factor as the participant's status may change during the course of followup. Availability of these time-dependent data is limited for many of the subjects in this sample; the study protocol did not call for annual assessment of health history until the implementation of the UDS in 2005.

All forms of MCI, and dementia as well, reflect a heterogeneous (and not completely understood) group of diseases including AD, hippocampal sclerosis, dementia with Lewy bodies, and vascular dementia [34, 48]. This heterogeneity may help explain the lack of significant predictors, other than baseline hypertension, from MCI_CC_ to dementia. We currently lack sufficient sample size to study these dementias as separate entities, but we have recently initiated work that will facilitate future research on which factors influence transitions into dementia subtypes. Similarly, MCI_CC_ is treated as a single entity here despite its well-documented heterogeneity [49] because we lack sufficient sample size to study the individual subtypes, and it is quite possible that risk factors for transitions to each subtype are different.

Limitations of the current study include that the final outcome for many of the included subjects is unknown as they continue to be followed longitudinally. Additional followup may change the results observed here, though they have face validity. The generalizability of the results is also somewhat limited due to the sample's demographic and geographic homogeneity, which would not be replicated in a population-based sample, and the nature of the longitudinal study, which requires brain donation at death. The volunteers are highly motivated and highly educated, and the frequency of both family history of dementia and APOE-4 is higher than what would be observed in the general population. Biomarker data (i.e., blood, cerebrospinal fluid, and neuroimaging) are for the most part unavailable on these subjects, and studies that have investigated risk factors for transition from clinical MCI to dementia have largely been focused on biomarkers [50, 51]. Obtaining biomarkers is extremely expensive, however, and it has been reported that longitudinal neuropsychological testing data provides as good or better accuracy in predicting which clinical MCI cases will convert versus remaining stable [52]. Nevertheless, the recently published criteria for the diagnosis of MCI due to AD make extensive use of biomarker data [24], and this modeling technique will allow us to incorporate these data as they become available in the future.

Finally, a large portion of this University of Kentucky-based longitudinal cohort was not included in this study (*n* = 476). The decision to exclude all subjects not in the original study [22] was due to the fact that the model's power to detect risks is based on the number of events in the sample, not the number of subjects. The excluded subjects, who are relatively recent recruits with about four assessments on average, are unable to contribute events due to this abbreviated followup. Potential differences between included and excluded participants were assessed using standard parametric two-group comparisons. Included and excluded subjects compare favorably on distribution of sex, family history, APOE-4, history of hypertension at baseline, and time between assessments (data not shown). Although the excluded subjects were slightly older at baseline, the effect size is small (Cohen's *d* = −0.05). Excluded subjects also have lower education (*χ* ^2^ = 8.8, 2 df, *P* = 0.01). That the excluded subjects are slightly older and less educated reflects that recruitment goals were changed in 2005 in order to enroll older participants with lower education, and all of the included subjects in the present model were recruited prior to 2005.

## Figures and Tables

**Table 1:** Criteria for state classification.

State	Definition
Normal cognition	No cognitive test score more than 1.5 standard deviations (SD) below the age-adjusted mean; absence of MCI_CC_ or Dementia (see below)

Test-based amnestic MCI (aMCI_TB_)	At least one score more than 1.5 SD below the age-adjusted mean on the following measures of episodic memory: Wechsler Logical Memory, Benton Visual Retention Test (number correct or number of errors), a word list (Consortium to Establish a Registry in Alzheimer's Disease word list or California Verbal Learning Test ) total learning score, delayed recall score, savings score, and the maximum recalled minus delayed recall score

Test-based mixed MCI (mMCI_TB_)	At least one score more than 1.5 SD below the age-adjusted mean on the following measures of language and executive function: phonemic or category verbal fluency, Boston Naming Test (15-item), and Trail Making Tests A or B

Clinical consensus-based MCI (MCI_CC_)	A cognitive complaint by the subject or informant, or evidence for longitudinal decline on cognitive test performance (at least 1.5 SD decline); generally intact global cognition; no or minimal functional impairment; not demented by DSM-IV criteria; neurodegenerative etiology suspected

Dementia	Meeting DSM-IV criteria for dementia, or NINCDS/ARDRA criteria for possible or probable AD, or NINDS-AIREN criteria for possible or probable vascular dementia, or DLB Consortium criteria for Lewy body disease

**Table 2:** Subject characteristics (*n* = 554).

Characteristic	Summary
Age at entry, *y* (mean ± SD)	72.7 ± 7.8
Female, %	64.3
Family history of dementia, %	41.3
At least one APOE-4 allele, %	30.0
>12 years of education, %	88.1
History of hypertension at entry, %	36.6
Hypertension treated with medication, %	86.5
Number of assessments (mean ± SD)	10.8 ± 4.5
Time between assessments, *y* (mean ± SD)	1.1 ± 0.4

**Table 3:** One-step transition matrix (number of assessments [% of prior visit state]).

Prior visit	Current visit					
	Normal	Amnestic MCI_TB_	Mixed MCI_TB_	Clinical Consensus MCI	Dementia	Death
Normal	2192 (68.3)	478 (14.9)	385 (12.0)	34 (1.1)	19 (0.6)	100 (3.1)
Amnestic MCI_TB_	448 (59.3)	148 (19.6)	108 (14.3)	23 (3.1)	10 (1.3)	18 (2.4)
Mixed MCI_TB_	341 (33.0)	88 (8.5)	453 (43.8)	47 (4.5)	27 (2.6)	79 (7.6)
Clinical Consensus MCI				101 (66.9)	34 (22.5)	16 (10.6)

**Table 4:** Estimated relative risks and 95% confidence intervals for one-step transitions to test-based amnestic MCI (aMCI_TB_), test-based mixed MCI (mMCI_TB_), or clinical consensus MCI (MCI_CC_) versus the base state of normal cognition (bolding denotes statistical significance).

Risk factor*	aMCI_TB_ versus Normal	mMCI_TB_ versus Normal	MCI_CC_ versus Normal
Age	**1.02 (1.01–1.04)**	1.07 (1.05–1.08)	**1.12 (1.09–1.15)**
Female sex (versus male)	**0.77 (0.62–0.95)**	1.01 (0.82–1.24)	0.71 (0.46–1.09)
Family history of dementia (yes versus no)	0.81 (0.65–1.00)	**0.76 (0.62–0.94)**	1.04 (0.66–1.64)
≥one APOE-4 allele (versus none)	1.04 (0.83–1.31)	1.12 (0.89–1.40)	**1.89 (1.21–2.95)**
≤12 years of education (versus >12 years)	1.24 (0.89–1.74)	1.79 (1.33–2.42)	**2.20 (1.24–3.91)**
History of hypertension (yes versus no)	0.95 (0.76–1.18)	1.04 (0.84–1.28)	0.79 (0.42–1.49)
aMCI_TB_ at prior assessment (versus normal)	1.15 (0.91–1.45)	1.00 (0.77–1.29)	**2.28 (1.30–4.00)**
mMCI_TB_ at prior assessment (versus normal)	0.76 (0.57–1.02)	**4.51 (3.63–5.61)**	**4.80 (2.94–7.81)**

*As risk factors do not depend on the prior state, covariate effects are the same regardless of whether transitions occur from a prior state of normal cognition, aMCI_TB_, or mMCI_TB_.

**Table 5:** Estimated relative risks and 95% confidence intervals for one-step transitions to dementia or death without dementia versus the base state of normal cognition or clinical consensus MCI (MCI_CC_) (bolding denotes statistical significance).

Risk factors* (normal is base state; no history of MCI_CC_)	Dementia versus normal	Death versus normal
Age	**1.19 (1.14–1.24)**	**1.18 (1.15–1.21)**
Female sex (versus male)	1.87 (0.95–3.68)	**0.68 (0.49–0.95)**
Family history of dementia (yes versus no)	1.66 (0.92–3.01)	0.82 (0.57–1.17)
≥one APOE-4 allele (versus none)	**2.33 (1.28–4.23)**	0.97 (0.67 –1.42)
≤12 years of education (versus >12 years)	0.75 (0.26–2.18)	1.33 (0.80 –.22)
History of hypertension (yes versus no)	0.79 (0.42–1.49)	**1.49 (1.07–2.08)**
aMCI_TB_ at prior assessment (versus normal)	1.85 (0.82–4.21)	0.64 (0.38–1.08)
mMCI_TB_ at prior assessment (versus normal)	**4.90 (2.58–9.30)**	**2.67 (1.88–3.79)**

Risk factors (MCI_CC_is base state)	Dementia versus MCI_CC_	Death versus MCI_CC_

Age	1.05 (0.98–1.13)	1.03 (0.94–1.13)
Female sex (versus male)	1.75 (0.67–4.56)	1.15 (0.65–3.76)
Family history of dementia (yes versus no)	2.88 (0.95–8.72)	0.68 (0.15–3.03)
≥one APOE-4 allele (versus none)	0.69 (0.22–2.16)	2.33 (0.61–8.90)
≤12 years of education (versus >12 years)	0.97 (0.27–3.46)	0.55 (0.10–2.99)
History of hypertension (yes versus no)	**0.30 (0.10–0.93)**	0.70 (0.20–2.47)

*As risk factors depend only on the base state, covariate effects in the top half of the table are the same whether transitions occur from a prior state of normal cognition, aMCI_TB_, or mMCI_TB_.

## References

[1] Winblad B, Palmer K, Kivipelto M (2004). Mild cognitive impairment—beyond controversies, towards a consensus: report of the International Working Group on Mild Cognitive Impairment. *Journal of Internal Medicine*.

[2] Dubois B, Albert ML (2004). Amnestic MCI or prodromal Alzheimer’s disease?. *The Lancet Neurology*.

[3] Ritchie K, Artero S, Touchon J (2001). Classification criteria for mild cognitive impairment: a population-based validation study. *Neurology*.

[4] Petersen RC (2004). Mild cognitive impairment as a diagnostic entity. *Journal of Internal Medicine*.

[5] Petersen RC, Morris JC (2005). Mild cognitive impairment as a clinical entity and treatment target. *Archives of Neurology*.

[6] Palmer K, Fratiglioni L, Winblad B (2003). What is mild cognitive impairment? Variations in definitions and evolution of nondemented persons with cognitive impairment. *Acta Neurologica Scandinavica, Supplement*.

[7] Palmer K, Backman L, Winblad B, Fratiglioni L (2008). Mild cognitive impairment in the general population: occurrence and progression to alzheimer disease. *American Journal of Geriatric Psychiatry*.

[8] Busse A, Angermeyer MC, Riedel-Heller SG (2006). Progression of mild cognitive impairment to dementia: a challenge to current thinking. *British Journal of Psychiatry*.

[9] Gauthier S, Touchon J (2005). Mild cognitive impairment is not a clinical entity and should not be treated. *Archives of Neurology*.

[10] Guillozet AL, Weintraub S, Mash DC, Marsel Mesulam M (2003). Neurofibrillary tangles, amyloid, and memory in aging and mild cognitive impairment. *Archives of Neurology*.

[11] Markesbery WR, Schmitt FA, Kryscio RJ, Davis DG, Smith CD, Wekstein DR (2006). Neuropathologic substrate of mild cognitive impairment. *Archives of Neurology*.

[12] Ganguli M, Dodge HH, Shen C, DeKosky ST (2004). Mild cognitive impairment, amnestic type: an epidemiologic study. *Neurology*.

[13] Jicha GA, Abner E, Schmitt FA (2008). Clinical features of mild cognitive impairment differ in the research and tertiary clinic settings. *Dementia and Geriatric Cognitive Disorders*.

[14] Morris JC, Fulling D (1988). Early Alzheimer’s disease. Diagnostic considerations. *Archives of Neurology*.

[15] Stern Y (2003). The concept of cognitive reserve: a catalyst for research. *Journal of Clinical and Experimental Neuropsychology*.

[16] Schmitt FA, Davis DG, Wekstein DR, Smith CD, Ashford JW, Markesbery WR (2000). ’Preclinical’ AD revisited: neuropathology of cognitively normal older adults. *Neurology*.

[17] Mortimer JA (1997). Brain reserve and the clinical expression of Alzheimer’s disease. *Geriatrics*.

[18] Schmand B, Smit JH, Geerlings MI, Lindeboom J (1997). The effects of intelligence and education on the development of dementia. A test of the brain reserve hypothesis. *Psychological Medicine*.

[19] Morris JC, Weintraub S, Chui HC (2006). The Uniform Data Set (UDS): clinical and cognitive variables and descriptive data from Alzheimer disease centers. *Alzheimer Disease and Associated Disorders*.

[20] Mueller SG, Weiner MW, Thal LJ (2005). Ways toward an early diagnosis in Alzheimer’s disease: the Alzheimer’s Disease Neuroimaging Initiative (ADNI). *Alzheimer’s and Dementia*.

[21] Schmitt FA, Wetherby MMC, Wekstein DR, Dearth CMS, Markesbery WR (2001). Brain donation in normal aging: procedures, motivations, and donor characteristics from the Biologically Resilient Adults in Neurological Studies (BRAiNS) project. *Gerontologist*.

[22] Kryscio RJ, Schmitt FA, Salazar JC, Mendiondo MS, Markesbery WR (2006). Risk factors for transitions from normal to mild cognitive impairment and dementia. *Neurology*.

[23] Kryscio RJ, Schmitt FA, Mendiondo MS, Markesbery WR (2007). Hypertension as a risk factor for transitions from normal to dementia or mild cognitive impairment. *Research and Practice in Alzheimer’s Disease*.

[24] Albert MS, DeKosky ST, Dickson D (2011). The diagnosis of mild cognitive impairment due to Alzheimer's disease: recommendations from the National Institute on Aging-Alzheimer's Association workgroups on diagnostic guidelines for Alzheimer's disease. *Alzheimer's and Dementia*.

[25] (1994). *American Psychiatric Association: Diagnostic and Statistical Manual of Mental Disorders*.

[26] McKhann G, Drachman D, Folstein M (1984). Clinical diagnosis of Alzheimer's disease: report of the NINCDS-ADRDA work group under the auspices of Department of Health and Human Services Task Force on Alzheimer's disease. *Neurology*.

[27] Roman GC, Tatemichi TK, Erkinjuntti T (1993). Vascular dementia: diagnostic criteria for research studies: report of the NINDS-AIREN International Workshop. *Neurology*.

[28] McKeith IG, Dickson DW, Lowe J (2005). Diagnosis and management of dementia with Lewy bodies: third report of the DLB consortium. *Neurology*.

[29] Weintraub S, Salmon D, Mercaldo N (2009). The Alzheimer’s Disease Centers’ Uniform Data Set (UDS): the neuropsychologic test battery. *Alzheimer Disease and Associated Disorders*.

[30] Neumann PJ, Araki SS, Arcelus A (2001). Measuring Alzheimer’s disease progression with transition probabilities: estimates from CERAD. *Neurology*.

[31] Salazar JC, Schmitt FA, Yu L, Mendiondo MM, Kryscio RJ (2007). Shared random effects analysis of multi-state Markov models: application to a longitudinal study of transitions to dementia. *Statistics in Medicine*.

[32] Skrondal A, Rabe-Hesketh S (2003). Multilevel logistic regression for polytomous data and rankings. *Psychometrika*.

[33] SAS Institute Inc. (2000–2004). *SAS 9.1.2 Help and Documentation: PROC NLMIXED*.

[34] Jicha GA, Abner EL, Schmitt FA (2012). Preclinical AD Workgroup staging: pathological correlates and potential challenges. *Neurobiology of Aging*.

[35] Portet F, Ousset PJ, Visser PJ (2006). Mild cognitive impairment (MCI) in medical practice: a critical review of the concept and new diagnostic procedure. Report of the MCI Working Group of the European Consortium on Alzheimer’s Disease. *Journal of Neurology, Neurosurgery and Psychiatry*.

[36] Fisk JD, Rockwood K (2005). Outcomes of incident mild cognitive impairment in relation to case definition. *Journal of Neurology, Neurosurgery and Psychiatry*.

[37] Fisk JD, Merry HR, Rockwood K (2003). Variations in case definition affect prevalence but not outcomes of mild cognitive impairment. *Neurology*.

[38] Brainerd CJ, Reyna VF, Petersen RC, Smith GE, Taub ES (2011). Is the apolipoprotein e genotype a biomarker for mild cognitive impairment? Findings from a nationally representative study. *Neuropsychology*.

[39] Boyle PA, Buchman AS, Wilson RS, Kelly JF, Bennett DA (2010). The APOE *ε*4 allele is associated with incident mild cognitive impairment among community-dwelling older persons. *Neuroepidemiology*.

[40] Tschanz JT, Corcoran CD, Schwartz S (2011). Progression of cognitive, functional, and neuropsychiatric symptom domains in a population cohort with alzheimer dementia: the cache county dementia progression study. *American Journal of Geriatric Psychiatry*.

[41] Fleisher AS, Sowell BB, Taylor C, Gamst AC, Petersen RC, Thal LJ (2007). Clinical predictors of progression to Alzheimer disease in amnestic mild cognitive impairment. *Neurology*.

[42] Brickman AM, Meier IB, Korgaonkar MS Testing the white matter retrogenesis hypothesis of cognitive aging.

[43] Elias MF, Sullivan LM, D’Agostino RB (2004). Framingham stroke risk profile and lowered cognitive performance. *Stroke*.

[44] Mungas D, Reed BR, Ellis WG, Jagust WJ (2001). The effects of age on rate of progression of Alzheimer disease and dementia with associated cerebrovascular disease. *Archives of Neurology*.

[45] Regan C, Katona C, Walker Z, Hooper J, Donovan J, Livingston G (2006). Relationship of vascular risk to the progression of Alzheimer disease. *Neurology*.

[46] Hoffman LB, Schmeidler J, Lesser GT (2009). Less Alzheimer disease neuropathology in medicated hypertensive than nonhypertensive persons. *Neurology*.

[47] Guan J-W, Huang C-Q, Li Y-H (2011). No association between hypertension and risk for Alzheimer's disease: a meta-analysis of longitudinal studies. *Journal of Alzheimer's Disease*.

[48] Nelson PT, Jicha GA, Schmitt FA (2007). Clinicopathologic correlations in a large Alzheimer disease center autopsy cohort: neuritic plaques and neurofibrillary tangles "do count" when staging disease severity. *Journal of Neuropathology and Experimental Neurology*.

[49] Hughes TF, Snitz BE, Ganguli M (2011). Should mild cognitive impairment be subtyped?. *Current Opinion in Psychiatry*.

[50] Risacher SL, Saykin AJ, West JD, Shen L, Firpi HA, McDonald BC (2009). Baseline MRI predictors of conversion from MCI to probable AD in the ADNI cohort. *Current Alzheimer Research*.

[51] Misra C, Fan Y, Davatzikos C (2009). Baseline and longitudinal patterns of brain atrophy in MCI patients, and their use in prediction of short-term conversion to AD: results from ADNI. *NeuroImage*.

[52] Cui Y, Liu B, Luo S (2011). Identification of conversion from mild cognitive impairment to alzheimer's disease using multivariate predictors. *PLoS One*.

